# Automated, cassette-based isolation and formulation of high-purity [^61^Cu]CuCl_2_ from solid Ni targets

**DOI:** 10.1186/s41181-020-00108-7

**Published:** 2020-11-05

**Authors:** Johan Svedjehed, Christopher J. Kutyreff, Jonathan W. Engle, Katherine Gagnon

**Affiliations:** 1 Cyclotrons and TRACERcenter, GE Healthcare, GEMS PET Systems AB, Uppsala, Sweden; 2 Department of Medical Physics, School of Medicine and Public Health, University of Wisconsin-Madison, Madison, WI USA; 3 Department of Radiology, School of Medicine and Public Health, University of Wisconsin-Madison, Madison, WI USA

**Keywords:** ^61^Cu (radiocopper), Automation/automated, PET, Solid target, Dissolution, Recycling

## Abstract

**Background:**

A need for improved, cassette-based automation of ^61^Cu separation from irradiated Ni targets was identified given the growing interest in theranostics, and generally lengthy separation chemistries for ^64^Cu/^64^Ni, upon which ^61^Cu chemistry is often based.

**Methods:**

A method for separating ^61^Cu from irradiated ^nat^Ni targets was therefore developed, with provision for target recycling. Following deuteron irradiation, electroplated ^nat^Ni targets were remotely transferred from the cyclotron and dissolved in acid. The dissolved target solution was then transferred to an automated FASTlab chemistry module, where sequential TBP and TK201 (Triskem) resins isolated the [^61^Cu]CuCl_2_, removed Ni, Co, and Fe, and concentrated the product into a formulation suitable for anticipated radiolabelling reactions.

**Results:**

^61^Cu saturation yields of 190 ± 33 MBq/μA from energetically thick ^nat^Ni targets were measured. The average, decay-corrected, activity-based dissolution efficiency was 97.5 ± 1.4% with an average radiochemical yield of 90.4 ± 3.2% (*N* = 5). The isolated activity was collected approximately 65 min post end of bombardment in ~ 2 mL of 0.06 M HCl (HCl concentration was verified by titration). Quality control of the isolated [^61^Cu]CuCl_2_ (*N* = 5) measured ^58^Co content of (8.3 ± 0.6) × 10^− 5^% vs. ^61^Cu by activity, Ni separation factors ≥ (2.2 ± 1.8) × 10^6^, EoB molar activities 85 ± 23 GBq/μmol and NOTA-based EoB apparent molar activities of 31 ± 8 MBq/nmol and 201 MBq/nmol for the 30 min and 3.3 h (*N* = 1) irradiations, respectively.

**Conclusion:**

High purity ^61^Cu was produced with the developed automated method using a single-use, cassette-based approach. It was also applicable for ^64^Cu, as demonstrated with a single proof-of-concept ^64^Ni target production run.

## Highlight statements

- A rapid, automated, and highly repeatable procedure for isolating [^61^Cu]CuCl_2_ from cyclotron-irradiated Ni targets was developed.
- The suitable formulation of the product facilitates labelling experiments.
- Enables routine production of ^61^Cu with NOTA-based AMAs of 200 MBq/nmol [5 Ci/μmol] from ^nat^Ni solid targets.

## Introduction

Within the field of positron emission tomography (PET), radiometals research has increased during the past decade (e.g. publications per year with the keyword ^68^Ga have increased from 69 to 506 between 2009 and 2019, Scopus). These radiometals complement the traditional PET nuclide ^18^F thanks to differences in their chemical and radioactive decay properties. This opens a path toward more personalised medicine as an array of biomolecules and biodistribution mechanisms can be used to adapt a treatment to a specific disease case. Radiometals are widely used for radiopharmaceuticals, in part due to available chelation chemistry and labelling with biomolecules (Krasikova et al. 2016; Price and Orvig 2014; Aluicio-Sarduy et al. 2018; Vivier et al. 2018; Tsai and Wu 2018; Liu 2008). Among the radiometals, following Ga, Cu is one of the most extensively investigated for PET radiopharmaceutical purposes (Mikolajczak et al. 2019; Brandt et al. 2018; Wadas et al. 2006; McCarthy et al. 1999). One reason for this is the well-understood coordination chemistry and biodistribution of Cu (Wadas et al. 2006; Wadas et al. 2010; Wallhaus et al. 1998; Jalilian et al. 2009; Woo et al. 2019), which has resulted in a multitude of chelators and biomolecule options being available for Cu isotopes.

While ^64^Cu has been suggested for theranostic applications, including pairing with ^177^Lu (Song et al. 2016), several Cu radioisotopes are suitable for both imaging and therapy. This creates an opportunity for Cu to be used as a “true” (i.e. identical element) theranostic pair: ^61^Cu (t_½_ = 3.34 h, 61% β^+^, E_Max_ = 1216 keV) is suitable for PET imaging; only 5.9 and 2.1% of its two major gammas (282.956 KeV, I_γ_ = 12.2%; 656.008 keV, I_γ_ = 10.8%, respectively), are coincident with β^+^ decay (IAEA 2020a). ^64^Cu (t_½_ = 12.7 h) is more commonly used in PET imaging (18% β^+^, E_Max_ = 653 keV) (Singh et al. 2017; Follacchio et al. 2017) with negligible gamma emissions (1345.77 keV, I_γ_ = 0.475%), however, applications in β^−^ and Auger emission therapy have been reported (39% β^−^, E_Max_ = 580 keV) (Obata et al. 2005; Lewis et al. 1999, 2001; Gutfilen et al. 2018), and ^67^Cu (t_½_ = 61.83 h, 100% β^−^, E_max_ = 562 keV) is a therapeutic radionuclide suitable for SPECT imaging (E_γ_ = 184.577 keV, I_γ_ = 48.7%).

^61^Cu and ^64^Cu share several physical properties; they are imageable with PET, have half-lives that allow for regional distribution and can both be made from Ni starting material. However, several production paths exist for these isotopes, and depending on the enrichment of the starting material, the production cost will vary significantly. Several ^61^Cu production routes start from Ni target materials, including the ^nat^Ni(d,x)^61^Cu, ^60^Ni(d,n)^61^Cu, and ^61^Ni(p,n)^61^Cu reactions, as seen in Table 1 alongside typical production routes for ^64^Cu and ^67^Cu. Each of these Ni-based ^61^Cu production routes, e.g. from ^nat^Ni to ^60^Ni to ^61^Ni, increase in theoretical thick target yields, but are met with increased cost of target material (estimated ~$1–2 USD/mg for ^60^Ni [nat ab. 26.223%], and ~ $30–40 USD/mg for ^61^Ni [nat ab. 1.1399%]). Enriched Ni options for producing ^64^Cu are limited to ^64^Ni (~$30–40 USD/mg for ^64^Ni [nat ab. 0.9255%]) through ^64^Ni(p,n)^64^Cu. This results in a possible lower production cost for ^61^Cu, compared with ^64^Cu, by using less expensive enriched options, at the cost of lower theoretical thick target yields.

**Table 1:** Common production paths and decay properties of ^61^Cu, ^64^Cu and ^67^Cu. All nuclear data was acquired from NuDat (Brookhaven National Laboratory 2020a) and Qcalc (Brookhaven National Laboratory 2020b). “Q” notes q-value

Isotope	Half-life	Decay mode	Nuclear reaction (MeV)	Nat. Ab. (%)
^61^Cu	3.34 h	β^+^ (61%) β^+^_mean_ = 500 keV β^+^_max_ = 1216 keV	^nat^Ni(d,x)^61^Cu ^60^Ni(d,n)^61^Cu (Q = 2.575) ^61^Ni(p,n)^61^Cu (Q = − 3.020) ^64^Zn(p,α)^61^Cu (Q = 0.844)	N/A 26.223 1.1399 49.17
^64^Cu	12.70 h	β^−^ (38.5%) β^−^_mean_ = 191 keV β^−^_max_ = 580 keV β^+^ (17.60%) β^+^_mean_ = 278 keV β^+^_max_ = 653 keV	^64^Ni(p,n)^64^Cu (Q = − 2.457) ^68^Zn(p,αn)^64^Cu (Q = − 7.790)	0.9255 18.45
^67^Cu	61.83 h	β^−^ (100%) β^−^_mean_ = 141 keV β^−^_max_ = 562 keV	^70^Zn(p,α)^67^Cu (Q = 2.619) ^64^Ni(α,p)^67^Cu (Q = − 4.644) ^68^Zn(γ,p)^67^Cu (Q = − 9.977)	0.61 0.9255 18.45

The relatively long, but different, half-lives of ^61/64^Cu provide an opportunity to study biodistributions of larger molecules with slower kinetics, such as peptides or antibodies (with ^61^Cu perhaps better suited for same-day imaging and ^64^Cu allowing for later time-point imaging). However, their application in PET imaging will depend on the purpose of the study, as ^61^Cu has a higher sensitivity, i.e. 3.43 vs 0.98 cps/Bq/mL (Williams et al. 2005), but slightly lower spatial resolution compared with ^64^Cu (Williams et al. 2005). The physical decay properties of ^64^Cu can also result in a relatively larger effective dose compared with ^61^Cu. E.g. the effective dose for [^64^Cu]Cu-PTSM and [^61^Cu]Cu-PTSM as perfusion imaging agents were 3.8 vs 2.5 mSv per 100 MBq respectively, according to Williams et al. (2005). Thus, ^61^Cu and ^64^Cu are both valuable diagnostic radionuclides whose applications should be tailored to their physical decay characteristics. However, relatively more papers are published on ^64^Cu than on ^61^Cu; 1288 vs 113 hits between 2009 and 2019 for the keywords ^64^Cu and ^61^Cu respectively (Scopus) – which likely arises from simplified, distribution-friendly ^64^Cu logistics. Nevertheless, the increased availability of cyclotron production facilities, increasing solid target infrastructure, and automated radiochemistry systems compel reconsideration of the utility of ^61^Cu.

Additionally, regardless of the production route, several Co radioisotopic contaminants will be produced, with ^55^Co, ^57^Co and ^58^Co being of greatest interest due to their relatively long half-lives (^55^Co t_½_ = 17.5 h, ^57^Co t_½_ = 272 d, ^58^Co t_½_ = 71 d). Their quantities will depend on the selected reaction, irradiation conditions, target thickness, and isotopic abundance of Ni in the target material. Consequently, in addition to separating Cu from the stable Ni target material, efficient separation of Co is also necessary. As enriched Ni may be cost-prohibitive to implement as single-use, especially ^61^Ni, target recycling is imperative. Efficient (> 96%) recovery and re-plating processes have been described (Avila-Rodriguez et al. 2007). For this reason, though ^61^Ni targets were not employed in this study, target recovery/recycling was also investigated, including a preliminary production using ^64^Ni.

This paper focuses on obtaining high-purity ^61^Cu via a cassette-based automated separation method using a two-column approach implemented on the FASTlab chemistry platform. A proof-of-concept ^64^Cu production (*N* = 1) was performed to demonstrate applicability of this method to the ^64^Ni(p,n)^64^Cu reaction. This is interesting as [^64^Cu]Cu-DOTA-TATE has recently been granted FastTrack review by the US FDA^1^ and has been determined to produce more true-positive lesion detections for neuroendocrine tumours than [^68^Ga]Ga-DOTA-TOC (Johnbeck et al. 2017). Regardless of radioisotope, the Cu product must consistently be of high radionuclidic purity with a high apparent molar activity (AMA). Thus, it is important to have a robust separation chemistry and rigorous quality control (QC) process to ensure the high quality of the product.

## Materials and methods

### Bench-top pre-studies

Prior to irradiating electroplated Ni, target dissolution and chemical separation processes were investigated at the bench using a heater block and FASTlab. Dissolution studies, initially on Ni foil, probed the effects of acid concentration, H_2_O_2_ to HCl ratio, and temperature on dissolution efficiency, speed, and compatibility with downstream separation chemistry. Separation studies focused on minimizing Ni, Co, and Fe in the final product. These separation studies were performed by spiking dissolved stable Ni with ppm Cu, Co, and Fe, and analysing samples via semi-quantitative colorimetric tests (such as Merck’s Mquant® colorimetric Ni kit, part number 1.14420, which allow for sub-ppm analysis), or, with low activity (kBq) spikes of ^61^Cu and ^55^Co, produced by proton (≤ 5 μAmin) and deuteron (≤ 5 μAmin) irradiations of ^nat^Ni foil. During the benchtop tests, the relative distribution of ^61^Cu (and similarly ^55^Co) were determined for the collected fractions with an Ortec LaBr (digibase, brilliance 380) gamma spectrometer.

### Target preparation and recycling

Electroplated targets ranging from 70 to 120 mg were prepared by first dissolving natural Ni powder (Alfa Aesar, 99.8%, 325 mesh) in 2 mL of concentrated HNO_3_ (Optima Grade, Fisher Chemical) and drying down under N_2_ gas flow at 85 °C. Next, the dried Ni was prepared into an electroplating solution following the method of Piel et al. (1992) to electrodeposit Ni onto gold plating substrates. We adapted this method to electrodeposit onto 99.9% silver plating substrates (10 mm deposited diameter). Briefly, the dried Ni was reconstituted in 2.3 mL of 2.4 M H_2_SO_4_ (made from concentrated, Optima Grade H_2_SO_4_, Fisher Scientific, and 18 MΩ-cm milli-Q water) and the pH of the solution was brought to ~ 9.1 using ~ 2.5 mL concentrated NH_4_OH (28%, Optima Grade, Fisher Scientific). To the pH-adjusted solution 250–300 mg of (NH_4_)_2_SO_4_ (99.9999%, Puratronic, Alfa Aesar) was added, and the solution was quantitatively transferred to an electrolytic cell. With a platinum wire cathode and laboratory DC power supply, constant currents of 40–90 mA were tested for optimization purposes, with a voltage of 6–7.5 V applied through the static electrochemical cell for 1–4 days.

A similar setup was used for re-plating of targets following irradiation. Namely, the Ni collection fraction following purification was then dried down under N_2_ gas flow. As above with the fresh target material, 2 mL of concentrated HNO_3_ was added and the solution dried again. The dried, recycled Ni was then electroplated on a silver substrate as above for subsequent irradiation. The ^64^Ni (84.8 mg) was electroplated on a gold plating substrate, according to the method of Piel et al. (1992) directly.

### Target irradiation

Electroplated, ^nat^Ni targets were irradiated with 8.4 MeV deuterons on a PETtrace 800 cyclotron (GE Healthcare) equipped with a QIS (ARTMS) automated target handling system with typical beam currents of 20–30 μA, and typical irradiation times being either ≤30 min for initial tests (*N* = 3), or, up to 3.3 h (i.e. one half-life) for scaled-up demonstration (*N* = 1). To enable recycling comparison, both 1× recycled targets (*N* = 3) and 2× recycled targets (*N* = 2) were evaluated. For the preliminary enriched target production, the ^64^Ni was irradiated with nominally 13.1 MeV protons at 20 μA for 1 hour. These experiments are summarized in Table 2.

**Table 2:** Information compilation of the ten electroplated target irradiations

N	Target prepared from recycled Ni	Irradiation time	Current (μA)	Purpose
3	No	10–25 min	10–20	Optimization
1	No	3.3 h	30	Scaled up demonstration
3	Yes, once	30 min	30	Recycling evaluation
2	Yes, twice	30 min	30	Recycling evaluation
1	Yes, many^a^	60 min	20	Enriched material evaluation

^a^Based on previous implemented chemistry, not used multiple times for this study

For optimisation purposes, ^nat^Ni targets were additionally irradiated for approximately 1 min with 1 μA of protons to produce a radiocobalt tracer via the ^nat^Ni(p,x)^55^Co reaction. However, for quantitative analysis, ^58^Co was used due to its longer half-life compared to ^55^Co (^58^Co t_½_ = 71 d, ^55^Co t_½_ = 17.5 h), as ^58^Co will be predominantly formed through the ^60^Ni(d,α)^58^Co reaction.

### Dissolution

Following automated transportation of the irradiated ^nat^Ni target from the cyclotron to the hot cell docking station, the target capsule was transferred to the QIS dissolution unit with tongs. Based on preliminary bench experiments, dissolution was in 3 mL 1:1 7 M HCl (Ultrapur, Merck): 30% H_2_O_2_ (ultratrace analysis, Merck) whereby H_2_O_2_ was added both to improve the dissolution and to oxidize Fe ions to Fe^3+^. These two reagents were mixed on-line and circulated over the target surface at 2 mL/min for ~ 23 min at an estimated solution temperature of ~ 60 °C (based on a heater sleeve set point of 111 °C and probing of the heated capsule exterior with a thermocouple). Finally, 3 mL 11.1 M HCl was automatically added to the dissolution solution, with 90 s bubbling with air to mix. Prior to transferring the solution to the FASTlab at 1 mL/min, the sequence was momentarily paused (*N* = 5), and ~ 200 μL of the nominal 6 mL solution was removed for pre-purification analysis. The irradiated ^64^Ni target was manually dissolved in a benchtop dissolution block and transferred to the FASTlab using a peristaltic pump at 1 mL/min, as with the automated target handling.

### [^61^Cu]CuCl_2_ separation

Separation was implemented on a cassette-based FASTlab platform using a 1 mL TBP column (a tributyl-phosphate-based resin, particle size 50–100 μm, pre-packed, Triskem, Britany, Fr) and 2 mL TK201 column (a tertiary-amine-based weak ionic exchange resin containing a small amount of a long-chained alcohol, particle size 50–100 μm, pre-packed, Triskem, Brittany, Fr) automatically conditioned in series with 7 mL H_2_O and 6 mL 11.1 M HCl. The cassette reagent vials were prepared using concentrated HCl (Optima Grade, Fischer Scientific), NaCl (ACS, Fischer Scientific) and milli-Q water (Millipore system, 18 MΩ-cm resistivity).

**Fig. 1:**
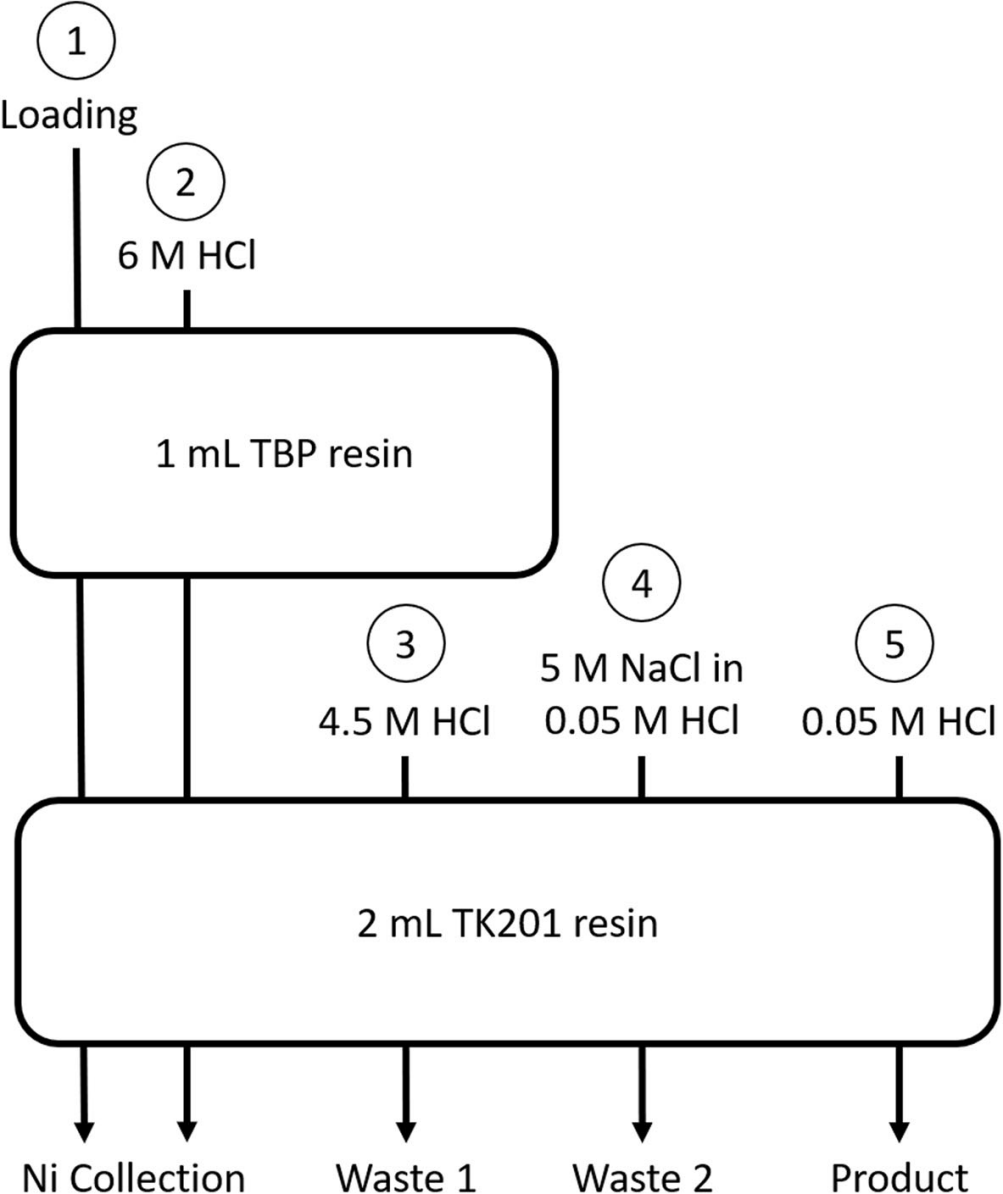
Two-column approach for ^61^Cu separation. Process steps described below

A general schematic of the resin loading, washing, and elution steps is given in Fig. 1, with detailed process steps described below:

Process steps:

1. The acid-adjusted dissolution solution (approx. 6 mL) was loaded over both columns in series and directed into a “Ni collection vial”. The TBP resin acted as a guard column as it quantitatively retained Fe^3+^ ions, while the Cu and Co chloride complexes were quantitatively retained on the TK201 resin.
2. Both columns were washed with 4 mL 6 M HCl to maximize Ni recovery for future recycling.
3. The TK201 column was washed with 5.5 mL 4.5 M HCl to elute the majority of Co (Waste #1)
4. The TK201 column was washed with 4 mL of 5 M NaCl in 0.05 M HCl to decrease residual acid on the resin and further remove any Co (Waste #2). In the longer term, Waste 1 and Waste 2 will be combined, but were separated for this study for analytical/optimization purposes.
5. The TK201 column was washed with 2 mL of 0.05 M HCl to quantitatively elute the [^61^Cu]CuCl_2_

### Analysis

#### Gamma ray spectrometry

For the electroplated target irradiations, ^61^Cu and ^58^Co activities were quantified by high purity germanium (HPGe) gamma spectrometry with an Al-windowed Canberra Model GC1519 (15% relative efficiency, full-width at half-maximum at 1173 keV = 1.8 keV) and used to determine the distribution in the samples and fractions. The gammas used for analysis were: ^58^Co (810.759 keV, I_γ_ = 99.45%) and ^61^Cu (primarily 282.956 keV, I_γ_ = 12.2% and 656.008 keV, I_γ_ = 10.8%). Samples were counted at a range of distances to the front face of the cylindrical detector body and were selected to maintain the dead time to ≤15%. The energy and efficiency calibration of the detector were performed using a 5-source method: ^241^Am, ^133^Ba, ^137^Cs, ^152^Eu, and ^60^Co.

#### Microwave plasma atomic emission spectrometry

Trace metal standards for Co, Cu, Fe, Ni, and Zn (1000 mg/L) were purchased from Sigma Aldrich. Trace metal analysis was conducted on aliquots of the collected sample fractions using an Agilent Technologies Model 4200 Microwave Plasma Atomic Emission Spectrometer (MP-AES). The concentration of HCl in each analysed sample aliquot was adjusted to 0.5 M. Calibration standards of 10, 50, 100, and 500 ppb and 1, 5, 10, and 50 ppm concentrations containing Co, Cu, Fe, Ni, and Zn were prepared in 0.5 M HCl and quantified using two atomic emission wavelengths for analysis of each element. The optimum wavelengths, depending on signal intensities, limits of detection, standard deviations and obvious interferants, were determined for the different elements post analysis.

The molar activity (MA) and separation factors (SF) of/for the [^61^Cu]CuCl_2_ product can be calculated from MP-AES quantified stable Cu and Ni. However, when assessing the separation factors, some samples contained Ni in concentrations below the method detection limit (MDL). When this was the case, our calculations assumed the MDL of 10 ppb as a conservative estimate of the Ni quantity.

#### Apparent molar activity

The apparent molar activity (AMA) was determined by titration with NOTA and adapted from the process described by McCarthy et al. (1997). Namely, 500 μL of [^61^Cu]CuCl_2_ was added to 0.6 mL 0.25 M Sodium acetate (anhydrous, 99% pure, Fisher Scientific), a solution pH of 4–5 was verified using Whatman pH strips, and the solution was vortexed. Next, 100 μL of this activity mixture was added to each of ten vials pre-loaded with both 40 μL 0.25 M sodium acetate (pH 4–5) and 100 μL of NOTA (~ 0.001–10 nmol). Samples were vortexed, individually assayed for activity, and incubated at room temperature for 15 min. Thin-layer chromatography (TLC) was performed by spotting each sample onto an aluminium-backed silica plate, developing in 1:1 MeOH:(10% w/v) NH_4_OAc and scanning on an OptiQuant autoradiography system (Perkin Elmer Cyclone Plus Storage Phosphor System). In plotting the sigmoidal curve of percent binding vs. NOTA concentration, the NOTA concentration required for 50% binding was identified. The AMA was then calculated as the average sample activity (decay corrected to EoB) and divided by 2× the NOTA concentration required for 50% binding.

#### Product HCl titration

To evaluate the suitability of the product formulation, and ensure residual HCl was minimized, titrations were performed to determine the HCl concentration of the product fraction. To this end, 0.5 mL of product fraction (*N* = 5) was added to approximately 10 mL milli-Q water with 100 μL of phenolphthalein in an Erlenmeyer flask with magnetic stir bar, and 5.8 mM NaOH was added dropwise from a burette until a faint pink colour visually persisted in the solution.

## Results and discussion

### Yields: ^61^Cu and [^61^Cu]CuCl_2_

The overall ^61^Cu yield was assessed by assaying the activity of the isolated product, waste vials, Ni recovery vial, resins, and target plate post dissolution. This resulted in a saturation yield (± SD) of 207 ± 26 MBq/μA (*N* = 3) for the initial low current optimization runs and 190 ± 33 MBq/μA (*N* = 5) for the subsequent 1×/2 × −recycled material. No significant difference was noted in saturation yield of the ^61^Cu between initial low current and recycled target irradiations. These saturation yields are also a conservative estimate due to the likely presence of unquantified residual activity in the lines and manifold, and possible fractional intercept of the 10 mm diameter Ni plating by the deuteron beam. Nevertheless these saturation yields are considered to be in reasonable agreement with the reported saturation yield (IAEA 2020b) of 248 MBq/μA for 8.4 MeV deuterons on ^nat^Ni. With regards to chemical processing, the average activity-based dissolution efficiency was 97.5 ± 1.4% (*N* = 5) with an average radiochemical yield of 90.4 ± 3.2% (*N* = 5) from the separation and an average dissolution + separation processing time of 65 ± 3 min. Plating efficiencies of 96.0 ± 0.9% (*N* = 3) were demonstrated for the fresh ^nat^Ni targets onto silver backings, as calculated from the ^nat^Ni input and plating masses. For the recycled ^nat^Ni targets, overall recycling efficiencies, i.e. the percent of Ni recovered and re-plated, were collectively determined to be 88% and 92% respectively for the first and second round of recycling.

### Formulation

Select recent examples of [^6x^Cu]CuCl_2_ purification methods are given in Table 3. Many suffer from lack of automation or have final formulations in large volumes or high acid concentrations. A product with a large volume or high acid concentration may need large buffer quantities or potentially time-consuming reformulation steps which are also subject to transfer losses. The motivation of this work was to address these concerns by developing a fast, efficient, automated process with attractive final formulation qualities.

**Table 3:** Comparison of requirements for [^6x^Cu]CuCl_2_ separation vs. other recent literature [reagents for target preparation and recycling not considered]

	This study	(Ohya et al. 2016, 2019)	(Matarrese et al. 2010)	(Jalilian et al. 2007)	(Strangis and Lepera 2007)	(Thieme et al. 2012)
Product isotope	^61^Cu	^64^Cu	^60^^/64^Cu	^64^Cu	^61^Cu	^64^Cu
Starting material	^nat^Ni	^64^Ni	^60/64^Ni	^68^Zn	^nat^Ni	^64^Ni
Automated	Yes	Yes	Yes	No ^b^	Partial	Partial
Disposable cassette-based purification	Yes	No	No	No	No	No
Prep. Time	< 1 h	1 day	- ^a^	- ^a^	- ^a^	- ^a^
Process time (min)	65 ± 3	~ 150	≥ 42 ^b^	105	90–120	~ 200
Total process acid consumption	160 mmol	> 200 mmol	460 mmol	> 1000 mmol	- ^a^	> 85 ^b^
Organics	No	Acetone	No	No	No	No
Radiochemical yield (%)	90.4 ± 3.2	88 ± 3	95	95	25–48	> 90
Formulation as stated	2 mL, < 0.1 M HCl	Evaporated then reconstituted in ≥10 mL UPW	10 mL, 0.5 M HCl	50 mL, 2 M HCl	- ^a^	Evaporated then reconstituted in 0.4 mL, 0.01 M HCl

^a^could not be determined from article

^b^not explicitly stated, assumed from context

When stating formulation, one must not assume that the acid concentration of the product is identical to that used for elution. As such, we titrated the HCl concentration of the [^61^Cu]CuCl_2_ product to assess the formulation directly. The HCl concentration of the product fraction was assessed on a subset (*N* = 5) of the product Cu vials (i.e. from the recycled productions) and determined to be 0.057 ± 0.002 M. This low product HCl concentration may circumvent the need for further product processing, such as roto-evaporation and reconstitution. This, in combination with the small product volume (2 mL), facilitates downstream radiolabelling by reducing the need for buffering. As a result, we are able to plan radiolabelling chemistries on the same single-use cassette using the FASTlab radiochemistry system.

Compared with the works cited in Table 3, our separation method is relatively fast – surpassed only by that of Matarrese et al. (2010). However, to achieve adequate labelling conditions, the method of Matarrese et al. would require ~ 40× the buffer than what was used in this work. Our method and the method presented by Ohya et al. (2016, 2019) have similar radiochemical yields. However, their method requires evaporation and has a considerably longer preparation and processing time. Overall, compared with the other methods in Table 3, our automated cassette-based purification method generally has a shorter preparation and process time, lower reagent consumption and more suitable product formulation.

### Competing radiocobalt production

As mentioned above, various Co radioisotopes will be produced during irradiations of Ni. McCarthy et al. (1999) notes, for example, 0.05, 0.04, and 0.04% of produced ^58^Co activity relative to ^61^Cu for the ^nat^Ni(d,x)^61^Cu, enriched ^60^Ni(d,n)^61^Cu, and ^61^Ni(p,n)^61^Cu reactions, respectively, whereas Strangis and Lepera (2007) notes 0.11% and 0.27% of produced ^58^Co and ^56^Co, respectively, relative to ^61^Cu, for the ^nat^Ni(d,x)^61^Cu reaction. The production of different Co isotopes, and relative production vs. ^61^Cu will depend on various parameters, including the nuclear reaction, the particle irradiation energy and time, target thickness, and isotopic composition. For the 200 μL pre-processed aliquots of dissolved target solution assayed in this study, a pre-purified ^58^Co to ^61^Cu activity ratio of 0.0465 ± 0.0046% at EoB, when irradiating for 30 min at 30 μA was determined. This is in line with previous reports (McCarthy et al. 1999; Strangis and Lepera 2007).

**Fig. 2:**
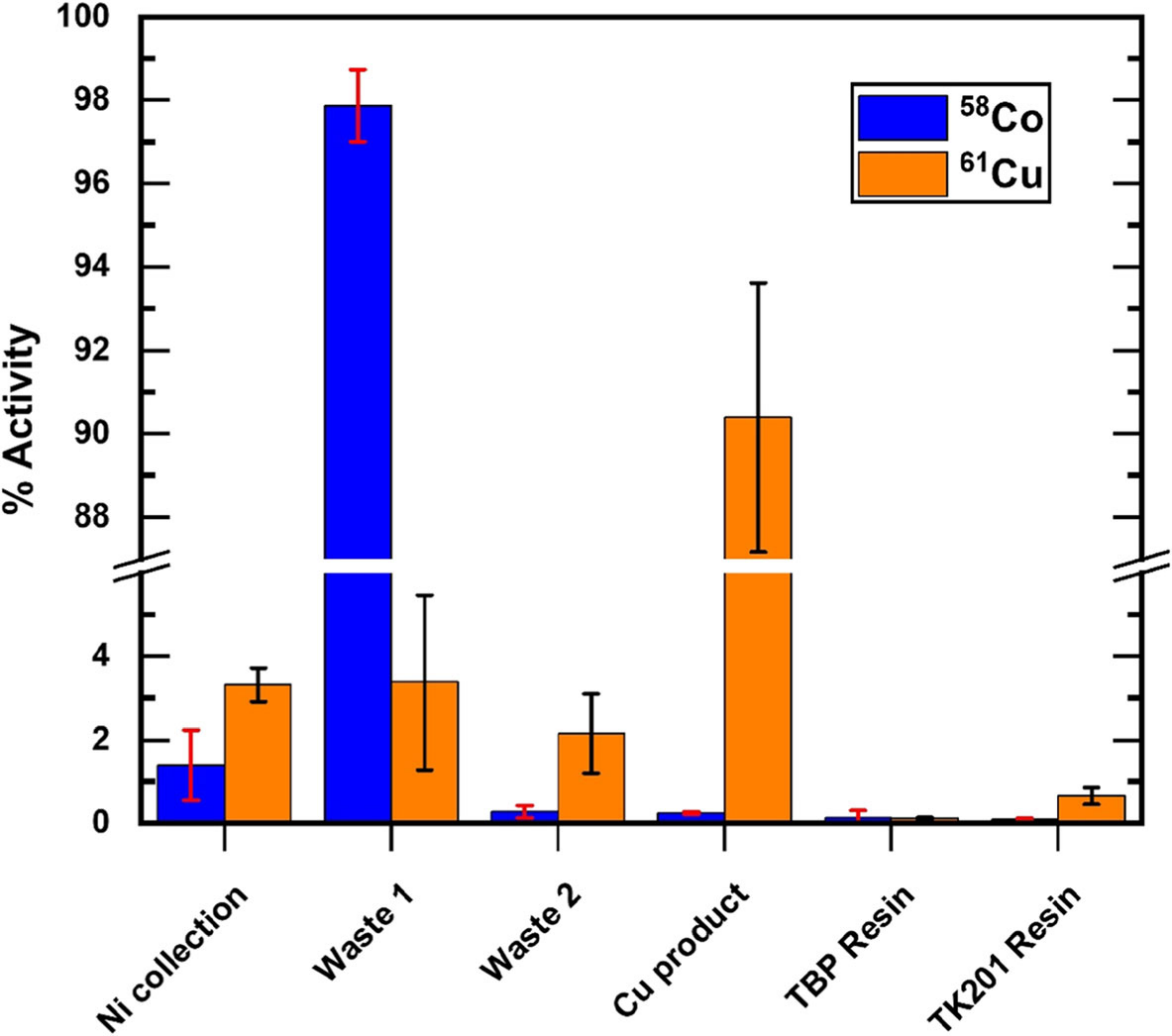
Bar diagram presenting the normalized activity distribution of ^58^Co and ^61^Cu. Note that the ^58^Co and ^61^Cu have been normalised individually

A radionuclidic impurity limit of 0.1% by activity at time of validity is currently noted in the European Pharmacopoeia for FDG (European Pharmacopoeia 2014), and (aside for ^66/67^Ga), for direct accelerator-produced ^68^Ga (European Pharmacopoeia 2020). Assuming a similar limit of ≤0.1% at time of validity for ^61^Cu, radiocobalt must be isolated from the ^61^Cu product to provide [^61^Cu]CuCl_2_ with a reasonable shelf-life. Measured ^58^Co and ^61^Cu content in the five recycled target separations are presented in Fig. 2 and Table 4 for the collected Ni/waste/production fractions, and resins. The distribution of each nuclide in Fig. 2 has been normalized individually. In this figure, we see that the majority of the ^58^Co (97.87 ± 0.86%) and ^61^Cu (90.4 ± 3.2%) are found in the waste and product vials, respectively. From an absolute perspective, the ^58^Co activity content in the purified [^61^Cu]CuCl_2_ product at EoB is (8.3 ± 0.6) × 10^− 5^%, resulting in a reduction of the ^58^Co to ^61^Cu ratio by more than a factor of 500 following purification.

**Table 4:** The normalized activity distribution data of ^58^Co and ^61^Cu (*N* = 5), which is visually represented in Fig. 2. Note that the ^58^Co and ^61^Cu have been normalised individually

Fraction	^61^Cu distribution (%)	^58^Co distribution (%)
Ni collection	3.31 ± 0.41	1.40 ± 0.84
Waste 1	3.4 ± 2.1	97.87 ± 0.86
Waste 2	2.2 ± 1.0	0.27 ± 0.15
Cu product	90.4 ± 3.2	0.245 ± 0.032
TBP resin	0.109 ± 0.028	0.13 ± 0.17
TK201 resin	0.66 ± 0.20	0.084 ± 0.032

### MAs, AMAs, and SFs

For the selected optimum wavelengths (Co 350 nm, Cu 324 nm, Fe 260 nm, Ni 352 nm and Zn 213 nm), the results of the MP-AES analysis largely generated concentrations below the method detection limit (MDL). For the analysed [^61^Cu]CuCl_2_ samples, only Cu produced consistent signals above the MDL, and the average product solution’s Cu concentration was determined to be 176 ± 37 ng/mL (*N* = 5). Co, Zn and Fe all produced signals < MDL (i.e. 100, 100, and 500 ppb respectively).

To assess the chemical purity and applicability of the product, MAs, AMAs, and Ni separation factors were determined and compiled, as reported in Table 5.

**Table 5:** Compilation of product MAs, AMAs and Ni separation factors for short and long irradiations

	^61^Cu Short irradiations (30 min) *N* = 5	^61^Cu Long irradiation (3.3 h) *N* = 1	^64^Cu Test irradiation (60 min) *N* = 1
MA (GBq/μmol) [Ci/μmol]	85 ± 23 [2.31 ± 0.62]^a^	Not measured	Not measured
NOTA AMA (GBq/μmol) [Ci/μmol]	31.3 ± 8.2 [0.85 ± 0.22]^a^	201 [5.43]^a^	179 [4.8]^a^
Ni separation factors	≥ (2.2 ± 1.8) × 10^6^	Not measured	Not measured

^a^at EoB

For longer irradiations, the MA is anticipated to increase proportionally with the produced activity, as the amount of stable Cu would not be expected to change significantly. Although not explicitly measured, if similar stable Cu (to the 30 min irradiations) is assumed for the 3.3 h irradiation, an estimated MA of 479 GBq/μmol [12.9 Ci/μmol] (N = 1) is calculated from the measured activity of that run at EoB. Additionally, assuming similar starting Ni quality, the MA would be expected to increase nearly 4-fold for enriched ^60^Ni targets. Therefore, MAs approaching 2 GBq/nmol [50 Ci/μmol] are not unrealistic for enriched ^60^Ni targets, and even higher MAs may be possible for enriched ^61^Ni irradiation. With this in mind, the results in Table 5 are promising, particularly when considering that the theoretical maximum MA for ^61^Cu is 35 GBq/nmol [939 Ci/μmol].

While a high MA is certainly desirable, it does not guarantee a chemically pure product capable of labelling, as other contaminants could be present and compete with the ^61^Cu for the chelator. A potentially more informative value is the AMA, which considers not only stable Cu, but also competing stable contaminants other than Cu. However, the AMA will depend on the chelator and labelling conditions, so comparing AMAs between different experimental setups is not straightforward.

### Proof of concept – ^64^Cu

To demonstrate applicability to ^64^Cu, a single test irradiation on enriched ^64^Ni was additionally performed. We report herein a NOTA-based AMA at EoB of 179 GBq/μmol [4.8 Ci/ μmol], and radiochemical yield of 93%, with an overall processing time of 65 min. Additionally, the ^64^Ni was recovered post-dissolution and re-plated, resulting in a recycling efficiency of 95%. This was on par with our noted ^nat^Ni recycling efficiencies between 88 and 92%.

## Conclusion

An automated method capable of producing high purity ^61^Cu from recycled ^nat^Ni targets has been developed. In summary, the product formulation is 2 mL of < 0.06 M HCl and the entire process can be achieved in ~ 65 min from EoB to EoS, with average radiochemical yields of 90.4 ± 3.2%, and AMAs > 5 Ci/μmol for NOTA when irradiating with 30 μA for 3.3 h. The [^61^Cu]CuCl_2_ isolation radiochemistry reported here has a measured separation factor ≥ (2.2 ± 1.8) × 10^6^ for Ni and ^58^Co radionuclidic impurity of < 0.0001% relative to ^61^Cu activity. Our process is also directly applicable to the production of ^64^Cu via the ^64^Ni(p,n)^64^Cu reaction, as was demonstrated in a preliminary, enriched ^64^Ni target irradiation and purification test. Additionally, the repeatable, single-use, cassette-based method is facile to introduce into GMP environments; its final ^61^Cu radiochemical yield and HCl concentration have relative standard deviations of 3.2 and 3.6%, respectively (*N* = 5). Finally, the method and FASTlab platform offer the possibility to perform radiolabelling on the same cassette as the separation.

## Data Availability

The datasets used and/or analysed during the current study are available from the corresponding author on reasonable request.

## Abbreviations

AMA: Apparent Molar Activity
MA: Molar Activity
MDL: Method Detection Limit
MP-AES: Microwave Plasma Atomic Emission Spectrometer
PET: Positron Emission Tomography
QC: Quality Control
SF: Separation Factor
TLC: Thin-layer Chromatography

## Footnotes

1 Granted FDA approval September 2020

## References

[CR1] Aluicio-Sarduy E, Ellison PA, Barnhart TE, Cai W, Nickles RJ, Engle JW (2018). PET radiometals for antibody labeling. J Label Compd Radiopharm.

[CR2] Avila-Rodriguez MA, Nye JA, Nickles RJ (2007). Simultaneous production of high specific activity ^64^Cu and ^61^Cu with 11.4 MeV protons on enriched ^64^Ni nuclei. Appl Radiat Isot.

[CR3] Brandt M, Cardinale J, Aulsebrook ML, Gasser G, Mindt TL (2018). An overview of PET radiochemistry, part 2: Radiometals. J Nucl Med.

[CR4] Brookhaven National Laboratory. NuDat [Internet]. 2020a [cited 2020 Jun 5]. Available from: https://www.nndc.bnl.gov/nudat2/.

[CR5] Brookhaven National Laboratory. QCalc [Internet]. 2020b [cited 2020 Jun 5]. Available from: https://www.nndc.bnl.gov/qcalc/.

[CR6] European Pharmacopoeia (2014). Fludeoxglucose (18F) injection.

[CR7] European Pharmacopoeia (2020). Monograph 01/2021:3109 gallium (^68^Ga) chloride (accelerator-produced) solution for radiolabelling.

[CR8] Follacchio GA, De Feo MS, De Vincentis G, Monteleone F, Liberatore M (2017). Radiopharmaceuticals labelled with copper radionuclides: clinical results in human beings. Curr Radiopharm.

[CR9] Gutfilen B, Souza SAL, Valentini G (2018). Copper-64: a real theranostic agent. Drug Des Devel Ther.

[CR10] IAEA. IAEA nuclear data section, Accessed 18 May 2020 [Internet]. 2020a [cited 2020 May 18]. Available from: https://nds.iaea.org/relnsd/vcharthtml/VChartHTML.html#lastnuc=61Cu.

[CR11] IAEA. IAEA nuclear data section, medical isotope browser, Accessed 5 May 2020 [Internet]. 2020b [cited 2020 May 18]. Available from: https://www-nds.iaea.org/relnsd/isotopia/isotopia.html.

[CR12] Jalilian AR, Rowshanfarzad P, Kamrani YY, Shafaii K, Mirzaii M (2007). Production and tumour uptake of [^64^Cu]pyruvaldehyde-bis (N 4-methylthiosemicarbazone) for PET and/or therapeutic purposes. Nucl Med Rev.

[CR13] Jalilian AR, Yousefnia H, Faghihi R, Akhlaghi M, Zandi H (2009). Preparation, quality control and biodistribution of [^61^Cu]-doxorubicin for PET imaging. Nucl Sci Tech.

[CR14] Johnbeck CB, Knigge U, Loft A, Berthelsen AK, Mortensen J, Oturai P (2017). Head-to-head comparison of ^64^Cu-DOTATATE and ^68^Ga-DOTATOC PET/CT: a prospective study of 59 patients with neuroendocrine tumors. J Nucl Med.

[CR15] Krasikova RN, Aliev RA, Kalmykov SN (2016). The next generation of positron emission tomography radiopharmaceuticals labeled with non-conventional radionuclides. Mendeleev Commun.

[CR16] Lewis JS, Laforest R, Buettner TL, Song SK, Fujibayashi Y, Connett JM (2001). Copper-64-diacetyl-bis(N4-methylthiosemicarbazone): an agent for radiotherapy. Proc Natl Acad Sci U S A.

[CR17] Lewis JS, Lewis MR, Cutler PD, Srinivasan A, Schmidt MA, Schwarz SW (1999). Radiotherapy and dosimetry of ^64^Cu-TETA-Tyr3-octreotate in a somatostatin receptor-positive, tumor-bearing rat model. Clin Cancer Res.

[CR18] Liu S (2008). Bifunctional coupling agents for radiolabeling of biomolecules and target-specific delivery of metallic radionuclides. Adv Drug Deliv Rev.

[CR19] Matarrese M, Bedeschi P, Scardaoni R, Sudati F, Savi A, Pepe A (2010). Automated production of copper radioisotopes and preparation of high specific activity [^64^Cu]Cu-ATSM for PET studies. Appl Radiat Isot.

[CR20] McCarthy DW, Bass LA, Cutler PD, Shefer RE, Klinkowstein RE, Herrero P (1999). High purity production and potential applications of copper-60 and copper-61. Nucl Med Biol.

[CR21] McCarthy DW, Shefer RE, Klinkowstien RE, Bass LA, Margeneau WH, Cutler CS (1997). Efficient production of high specific activity ^64^Cu using a biomedical cyclotron. Nucl Med Biol.

[CR22] Mikolajczak R, van der Meulen NP, Lapi SE (2019). Radiometals for imaging and theranostics, current production, and future perspectives. J Label Compd Radiopharm.

[CR23] Obata A, Kasamatsu S, Lewis JS, Furukawa T, Takamatsu S, Toyohara J (2005). Basic characterization of ^64^Cu-ATSM as a radiotherapy agent. Nucl Med Biol.

[CR24] Ohya T, Minegishi K, Suzuki H, Nagatsu K, Fukada M, Hanyu M (2019). Development of a remote purification apparatus with disposable evaporator for the routine production of high-quality ^64^Cu for clinical use. Appl Radiat Isot.

[CR25] Ohya T, Nagatsu K, Suzuki H, Fukada M, Minegishi K, Hanyu M (2016). Efficient preparation of high-quality ^64^Cu for routine use. Nucl Med Biol.

[CR26] Piel H, Qain SM, Stücklin G (1992). Excitation functions of (p,xn)-reactions on ^nat^Ni and highly enriched ^62^Ni: possibility of production of medically important radioisotope ^62^Cu at a small cyclotron. Radiochim Acta.

[CR27] Price EW, Orvig C (2014). Matching chelators to radiometals for radiopharmaceuticals. Chem Soc Rev.

[CR28] Singh A, Kulkarni HR, Baum RP (2017). Imaging of prostate cancer using ^64^Cu-labeled prostate-specific membrane antigen ligand. PET Clin.

[CR29] Song IH, Lee TS, Park YS, Lee JS, Lee BC, Moon BS (2016). Immuno-PET imaging and radioimmunotherapy of ^64^Cu−/^177^Lu-labeled anti-EGFR antibody in esophageal squamous cell carcinoma model. J Nucl Med.

[CR30] Strangis R, Lepera CG (2007). Production of ^61^Cu by deuteron irradiation of natural Ni. Proc 18th Int Conf Cyclotrons their Appl 2007. Cyclotrons.

[CR31] Thieme S, Walther M, Pietzsch HJ, Henniger J, Preusche S, Mäding P (2012). Module-assisted preparation of ^64^Cu with high specific activity. Appl Radiat Isot.

[CR32] Tsai WTK, Wu AM (2018). Aligning physics and physiology: engineering antibodies for radionuclide delivery. J Label Compd Radiopharm.

[CR33] Vivier D, Sharma SK, Zeglis BM (2018). Understanding the in vivo fate of radioimmunoconjugates for nuclear imaging. J Label Compd Radiopharm.

[CR34] Wadas T, Wong E, Weisman G, Anderson C (2006). Copper chelation chemistry and its role in copper radiopharmaceuticals. Curr Pharm Des.

[CR35] Wadas TJ, Wong EH, Weisman GR, Anderson CJ (2010). Coordinating radiometals of copper, gallium, indium, yttrium, and zirconium for PET and SPECT imaging of disease. Chem Rev.

[CR36] Wallhaus TR, Lacy J, Whang J, Green MA, Nickles RJ, Stone CK (1998). Human biodistribution and dosimetry of the PET perfusion agent copper- 62-PTSM. J Nucl Med.

[CR37] Williams HA, Robinson S, Julyan P, Zweit J, Hastings D (2005). A comparison of PET imaging characteristics of various copper radioisotopes. Eur J Nucl Med Mol Imaging.

[CR38] Woo SK, Jang SJ, Seo MJ, Park JH, Kim BS, Kim EJ (2019). Development of ^64^Cu-NOTA-trastuzumab for HER2 targeting: a radiopharmaceutical with improved pharmacokinetics for human studies. J Nucl Med.

